# Semantic processing of iconic signs is not automatic: Neural evidence from hearing non-signers

**DOI:** 10.1017/s1366728924001093

**Published:** 2025-02-10

**Authors:** Emily M. Akers, Katherine J. Midgley, Phillip J. Holcomb, Karen Emmorey

**Affiliations:** 1 Joint Doctoral Program in Language and Communicative Disorders, San Diego State University & University of California, San Diego; 2 Department of Psychology, San Diego State University; 3 School of Speech, Language, and Hearing Sciences, San Diego State University

**Keywords:** iconicity, American Sign Language, event-related potentials, N400, attention

## Abstract

Iconicity facilitates learning signs, but it is unknown whether recognition of meaning from the sign form occurs automatically. We recorded ERPs to highly iconic (transparent) and non-iconic ASL signs presented to one group who knew they would be taught signs (learners) and another group with no such expectations (non-learners). Participants watched sign videos and detected an occasional grooming gesture (no semantic processing required). Before sign onset, learners showed a greater frontal negativity compared to non-learners for both sign types, possibly due to greater motivation to attend to signs. During the N400 window, learners showed greater negativity to iconic than non-iconic signs, indicating more semantic processing for iconic signs. The non-learners showed a later and much weaker iconicity effect. The groups did not differ in task performance or in P3 amplitude. We conclude that comprehending the form-meaning mapping of highly iconic signs is not automatic and requires motivation and attention.

## 1. Introduction

People process words automatically and unconsciously in their native language. Evidence for automatic word recognition comes from both Stroop tasks and masked priming paradigms. In color-word Stroop tasks, participants must name the ink color of a word; however, participants automatically read the word, which interferes with the color-naming task when the word and color are different and facilitates color-naming when they are the same. (Stroop, 1935; Atkinson et al., 2003). Masked priming paradigms provide evidence for unconscious word processing because the prime word is presented subliminally (fast and masked) and yet still influences recognition of the target word, e.g., a reduced N400 for related compared to unrelated primetarget pairs (Holcomb & Grainger, 2006; Grainger et al., 2006). Evidence from bilingual studies indicates that the automaticity of word recognition is influenced by proficiency in each language. For example, Stroop effects are greater for the dominant language and are equal when a bilingual’s languages are balanced (Rosselli et al., 2002). In addition, Stroop effects increase with learning as language proficiency and use increase (Mägiste, 1984). Masked priming effects also increase with language experience (e.g., Sabourin et al., 2014). Thus, word processing becomes more automatic with learning and experience.

Co-speech gestures, like words, may also be processed automatically and unconsciously. For example, speech and gesture can be unintentionally combined into a single representation in memory, even when they convey different informations (Gurney et al., 2013; Johnstone et al., 2023). In this case, a misleading gesture can cause individuals (particularly children) to misremember when questioned about an event they witnessed, e.g., mis-recalling that a woman wore a striped (rather than a polka dot) dress when the interviewer produced a gesture indicating stripes while asking about the dress pattern (Johnstone et al., 2023). Similarly, additional information conveyed by gesture is automatically integrated into the meaning of a sentence, such that listeners incorporate gesture information during recall (Cassell et al., 1999; Kelly et al., 1999). For example, after watching a video of a woman saying, “my brother went to the gym” while producing a gesture depicting shooting a basketball, participants were more likely to report that the woman’s brother had gone to the gym to play basketball compared to participants who viewed the “no gesture” video (Kelly et al., 1999). These results suggest that both children and adults may automatically extract the meaning of the gestures they perceive.

One goal of the present study was to use event-related potentials (ERPs) to assess the hypothesis that adults automatically access the meaning of gestures. Rather than co-speech gestures, however, we presented signs from American Sign Language (ASL) that were highly transparent – the meaning was guessable by non-signers. For example, the sign DRINK^1^ (https://asl-lex.org/visualization/?sign=drink) resembles the act of drinking, and the meaning is transparent to non-signers (Sehyr & Emmorey, 2019). Wu and Coulson (2005) have proposed that meaningful gestures engage semantic processes that are analogous to those evoked by words. In their ERP study, participants made congruency judgments for a short cartoon clip followed by either a semantically congruent gesture (e.g., depicting the action shown in the cartoon) or an incongruent gesture (depicting a different action). Incongruent gestures elicited a larger N400-like component compared to congruent gestures. Similarly, Akers et al. (2024) found a larger N400 response when non-signers (prior to learning) made congruency judgments between an English word and a highly iconic (transparent) ASL sign – incongruent trials elicited greater negativity compared to congruent trials. This N400 priming effect was not observed for non-iconic signs that constituted meaningless gestures for the participants (before learning).

The tasks in both Wu and Coulson (2005) and Akers et al. (2024) required semantic processing because participants had to decide whether the gesture matched either a preceding cartoon or a preceding word. To our knowledge, no study has investigated whether iconic signs/gestures evoke meaning in sign-naïve people when the task does not explicitly promote meaning access. Whether automatic access to meaning occurs for highly iconic gestures when the task does not promote semantic processing is unclear. The current study addresses this question by using a probe task that does not require a semantic decision – detect an occasional grooming gesture, such as a person scratching their head.

The participants from Akers et al. (2024) had been recruited for an ASL learning experiment, and they performed a grooming gesture detection task prior to learning any ASL signs. This task preceded the word-sign matching task described above, which also occurred before learning the meaning of any signs. During the gesture detection task, participants were asked to respond whenever they saw an occasional grooming gesture among videos of highly iconic (meaningful) and non-iconic (meaningless) signs. These participants, because they knew that they would later be learning ASL signs and would be tested on their knowledge, can be considered highly motivated to extract meaning from the signs. To determine whether the motivation to learn ASL impacted how signs/gestures were processed prior to learning, we tested a separate group of participants who were not recruited for the ASL learning study and were considered to have low motivation to extract meaning from the signs. This second group was recruited immediately after they participated in other reading or picture processing ERP studies ongoing in the lab. These participants were only invited to complete the gesture detection task after they completed the study that they were originally recruited for. The seemingly offhand manner in which these non-learners were recruited served to reduce any chances for preparation or motivational expectations, as these participants had no expectation of learning or viewing any ASL signs. By comparing these two groups of participants, we were able to test (a) whether the expectation to learn influences the semantic processing of signs pre-learning and (b) whether meaning is automatically accessed from highly iconic signs when the task does not require semantic processing.

If participants are semantically processing highly iconic signs, we predict a larger N400 response (more negativity) compared to non-iconic signs because access to meaning has been shown to produce greater neural activity between 300 and 600 ms across a variety of stimulus types. For example, previous research has shown that when learners were tested throughout a semester, the amplitude of the N400 grew with more familiarization to the new second language words (Soskey et al., 2016). Transparent iconic signs may be processed as familiar gestures since their meaning is highly guessable. Crucially, if meaning processing is automatic (little attention needed), then both groups (learners and non-learners) should show an iconicity effect (iconic signs elicit more negativity than non-iconic signs). However, if a meaning-promoting task is required to engage semantic processing, then neither group is predicted to show an iconicity effect. Finally, if an intention or expectation to learn is critical to promote access to meaning, then we expect to only see an iconicity effect for the group of participants who were expecting to learn ASL.

## 2. Methods

### 2.1. Participants

Participants included 64 monolingual, native English speakers who did not know ASL (beyond the fingerspelled alphabet or a few isolated signs). Thirty-two were from Akers et al. (2024) and were recruited with the anticipation of learning ASL across three days (18 females; mean age 21 years, SD = 2.37, range = 18–27 years). These participants had not yet received the ASL training sessions reported in Akers et al. (2024) when they performed the grooming gesture detection task. However, these participants knew that they had been enrolled in a lab-learning experiment in which they would later be taught ASL signs over the course of a few days. The other 32 participants were recruited after they had already completed other unrelated ERP studies in our lab and therefore had no expectation of learning any ASL (24 females; mean age = 26, SD = 7.59, range = 19–50 years). All participants were right-handed, except one participant in the non-learner group who was left-handed, and all had either normal or corrected-to-normal vision. Participants reported no history of neurological disorders or learning impairments. Both groups of participants were drawn from the same population of young adults and were recruited from San Diego State University and the surrounding area. Data from an additional three participants in the non-learner group was collected and excluded – two misunderstood the task, and one was not a native English speaker.

All participants were treated in accordance with SDSU IRB guidelines. They were given informed consent and were given monetary compensation for participation.

### 2.2. Stimuli

The stimuli consisted of 100 video clips of ASL signs (from Akers et al., 2024) and 13 video clips of grooming gestures produced by the same native female signer. Videos were presented on an LCD video monitor while the participants sat 110 cm (43in) away from the screen. The video size was 10 × 13.25 cm in the center of the screen with a visual angle of 5.21 × 6.89 degrees. The signer was positioned in the middle of the frame so that her signing could be perceived without the participant needing to move their eyes. All videos started with the sign model in a resting position with her hands on her lap and ended when her hands returned to her lap. The average video length was 2157 ms (SD = 290 ms), with an average sign onset of 578 ms (SD = 104 ms). Sign onset was determined as in Caselli et al. (2017). Briefly, sign onset is defined as the first video frame that contains the fully formed handshape at its target location on the body or in signing space. The average grooming gesture video length was 3145 ms (SD = 379 ms), with an average gesture onset of 545 ms (SD = 114 ms). Examples of grooming gestures included the sign model rubbing her eyes, picking her fingernails, scratching her head and adjusting her clothing.

The 50 highly iconic signs from Akers et al. (2024) were selected based on iconicity ratings from the ASL-LEX database (http://asllex.org; Caselli et al., 2017; Sehyr et al., 2021). Iconicity ratings in this database were completed by hearing non-signers using a scale of 1 (not iconic) to 7 (very iconic) (see Caselli et al., 2017, for the full instructions for the iconicity ratings). The iconic signs all had ratings over 5.0 (M = 6.3, SD = .51). In addition, to ensure that the meanings of these iconic signs were transparent or “guessable,” Akers et al. (2024) utilized the transparency ratings from the ASL-LEX database and collected additional ratings when transparency information was not available in the database. Transparency was rated by hearing non-signers who were asked to guess the meaning of an ASL sign and then to rate how obvious their guessed meaning would be to others on a scale of 1 (not obvious at all) to 7 (very obvious). All iconic signs had a transparency rating of over 4.0 (M = 5.05, SD = .60). Examples of highly iconic, transparent signs are CIRCLE (https://asl-lex.org/visualization/?sign=circle) (index finger traces a circle in the air) and BRUSH (https://asl-lex.org/visualization/?sign=brush) (depicts brushing one’s hair). The average video length for the iconic signs was 2189 ms (SD = 330 ms), and the average sign onset within the video was 569 ms (SD = 97 ms).

The other 50 signs from Akers et al. (2024) were non-iconic with an average video length of 2124 ms (SD = 241 ms) and an average sign onset within the video of 587 ms (SD = 111 ms). These signs had an iconicity rating of under 3.0 (M = 1.92, SD = .47) and a transparency rating of under 4.0 (M = 3.37, SD = .34). Video links for all signs and videos of the grooming gestures are on the project’s OSF page (https://osf.io/7avju/).

### 2.3. Procedure

The ERP session consisted of a gesture detection task in which participants passively viewed the signs and pressed a button on a gamepad when they detected a grooming gesture. Participants were told that they would see videos of signs, and their task was to identify a video that looked like a gesture and not sign language, such as when the signer scratched her head or stretched out her arms (demonstrated by the experimenter). Both the learner and the non-learner groups received the same instructions.

**Figure 1.**
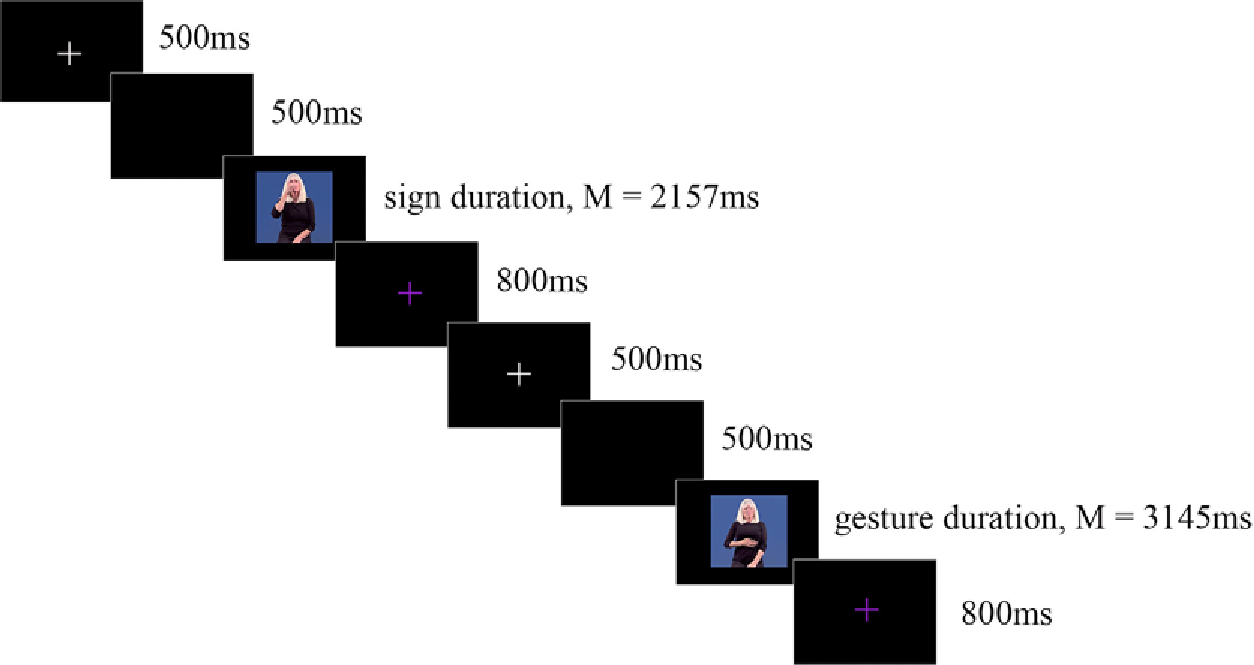
Schematic of the timing parameters for the gesture-detection task.

Each trial began with a white fixation cross for 500 ms followed by a blank screen for 500 ms. Immediately after the blank screen, a grooming gesture or a sign video was presented. After this, a trialending 800 ms purple fixation was displayed, indicating it was OK to blink before the beginning of the next trial. Participants were asked to respond as quickly and as accurately as they could (see Figure 1 for a schematic of a typical trial). All other stimuli (i.e., the ASL signs) did not require a button press.

There were two stimulus lists, which contained the same signs and gestures but in reverse presentation order. The lists were counterbalanced across participants. Both lists were pseudo-randomized so that no more than three trials in a row were in the same condition (iconic or non-iconic). Six additional signs (three iconic and three non-iconic) and two grooming gestures were used in a short practice session prior to the ERP session to introduce the task to the participants and to provide time for any questions. These trials were not included in the analyses.

### 2.4. EEG recording

All participants were seated in a comfortable chair in a darkened, sound-attenuating room. EEG was continuously recorded through a 29-channel cap with tin electrodes (Electro-cap International, Inc., Eaton, OH). There were four loose electrodes placed on the participant’s head at the following locations: one underneath the left eye to track blinking, one on the side of the right eye to track horizontal eye movements, and one placed on each mastoid bone behind the ear-- the left mastoid was used as the reference electrode, and the right was recorded actively. All electrodes were connected using a saline-based gel (Electro-Gel), and impedances were reduced to under 2.5 kΩ. The data was collected through Curry Data Acquisition software with a sampling rate of 500 Hz, and the EEG signal was amplified by a SynAmpsRT amplifier (Neuroscan-Compumedics, Charlotte, NC) with a bandpass of DC to 100 Hz.

### 2.5. Data analysis

ERPs were time-locked to the video onset with a 100 ms pre-stimulus baseline. Twelve electrode sites were analyzed to identify effects across a representative sample of scalp sites (F3, FZ, F4, C3, Cz, C4, P3, Pz, P4, O1, Oz and O2; see Supplementary Materials, Figure 1, for an illustration of the sites that were analyzed). Prior studies conducted in our lab have indicated that this grid-like analysis approach provides the best coverage of the scalp distribution, along with the fewest statistical comparisons (e.g., Yum et al., 2014). Following Akers et al. (2024), we focused on four main ERP epochs: 400–600 ms (transitional information leading up to sign onset for most signs), 600–800 ms (the earliest time window that could represent semantic processing based on the average sign onset), 800–1000 ms (expected N400 window based on average sign onset) and 1000–1400 ms (to track later effects known to happen in L2 learners).

To remove eye blinks and other eye artifacts prior to data analysis, independent component analysis (ICA) from the EEGLAB function under MATLAB was used (Makeig et al., 1996). These components were removed from the data prior to averaging (between one and three components were removed per participant). ERPs from individual sites were processed with a 15Hz low-pass filter prior to analysis. Trials that had artifacts post-ICA were removed from the analysis (post-ICA: learners = 0.47% trials rejected; non-learners = 0.22% trials rejected).

A mixed ANOVA design was used where Group (learners versus non-learners) was treated as a between-subjects variable and Iconicity (iconic versus non-iconic signs) and scalp distribution (Anteriority – frontal versus central versus parietal versus occipital; Laterality – left versus middle versus right) were treated as repeated measures (i.e., within-subject variables). For effects that showed a group difference, separate repeated measures analyses were performed as a function of Iconicity and the two scalp distributional factors for each group.

To determine whether there was a difference between the learners and non-learners in decision-making processes for the gesture detection task, we conducted a separate analysis examining the P3 component, comparing ERP responses to gestures and signs. For the P3 analysis, we compared the response to grooming gestures and iconic signs. We selected iconic signs for the comparison because both grooming gestures and iconic signs have potential meanings (e.g., scratching could convey boredom); however, the results were similar if non-iconic signs were used in the comparison. Only gesture “hits” were included in this analysis. We selected the time epoch of 800–1400 ms post-video onset to account for the range of sign onsets within the video. Since our average sign onset was 578 ms, 800 ms is roughly 300 ms post onset and 1400 ms is roughly 500 ms after the longest sign onset.

Significant results (*p* < .05) are reported below for the time windows of interest. Partial eta squared (η_p_^2^) is reported as a measure of effect size, and the Greenhouse and Geisser (1959) correction was used for all significant effects with a degree of freedom numerator greater than one.

## 3. Results

### 3.1. Behavioral results

There were no significant group differences in accuracy or false alarms for the gesture detection task (all *ps* > .57); see Table 1.

### 3.2. ERP results

**Figure 2.**
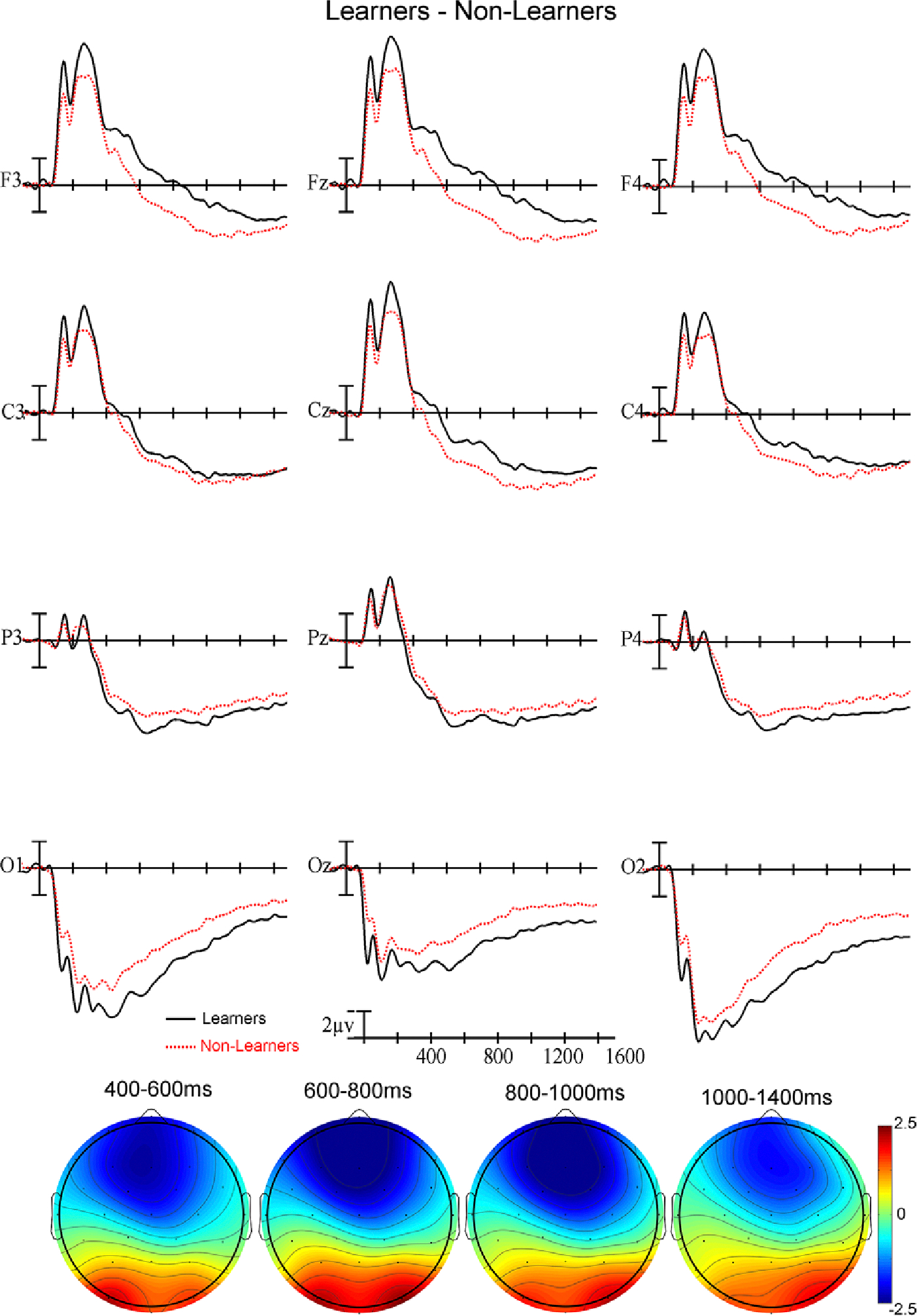
(Top) ERPs to all signs for learners and non-learners at the 12 electrode sites used in the ANOVAs. Negative is plotted up in this and all subsequent figures. (Bottom) Voltage maps formed by subtracting learners’ ERP trial data from non-learners’ ERP trial data in the four latency ranges.

Plotted in Figure 2 are the ERPs and voltage maps for all ASL signs (iconic and non-iconic combined) time-locked to the onset of the sign videos, and the learner group (black) and the non-learner group (red) are overplotted. As can be seen, the learners showed greater frontal negativity and greater posterior positivity compared to the non-learners throughout the recording, and this difference was most evident in the voltage maps for the analyzed epochs.

#### 400–600 ms time epoch.

In this early epoch, there was a significant interaction between Group and Anteriority (F(3,186) = 4.77, *p* = .0263, η_p_^2^ = .0715) – learners showed a greater anterior negativity and a greater posterior positivity compared to non-learners (see Figure 2). There were no interactions between Group and Iconicity in this epoch (all *ps* > .31).

#### 600–800 ms time epoch.

In this second epoch, there was a significant interaction between Group and Anteriority (F(3,186) = 8.08, *p* = .0032, η_p_^2^ = .1153) – the greater anterior negativity and posterior positivity for learners compared to non-learners continued in this epoch. Again, there were no significant interactions between Group and Iconicity (all *ps* > .16).

#### 800–1000 ms time epoch.

In this third epoch (~300–500 ms post sign onset), there was a significant main effect of Iconicity (F(1,62) = 5.37, *p* = .0238, η_p_^2^ = .0797), with iconic signs showing greater negativity than non-iconic signs. There was again a significant interaction between Group and Anteriority (F(3,186) = 7.34, *p* = .0047, η_p_^2^ = .1058), with learners showing greater anterior negativity and posterior positivity. In addition, there was a two-way interaction between Group and Iconicity (F(1,62) = 4.33, *p* = .0417, η_p_^2^ = .0652). Therefore, we ran separate follow-up ANOVAs on each group.

**Figure 3.**
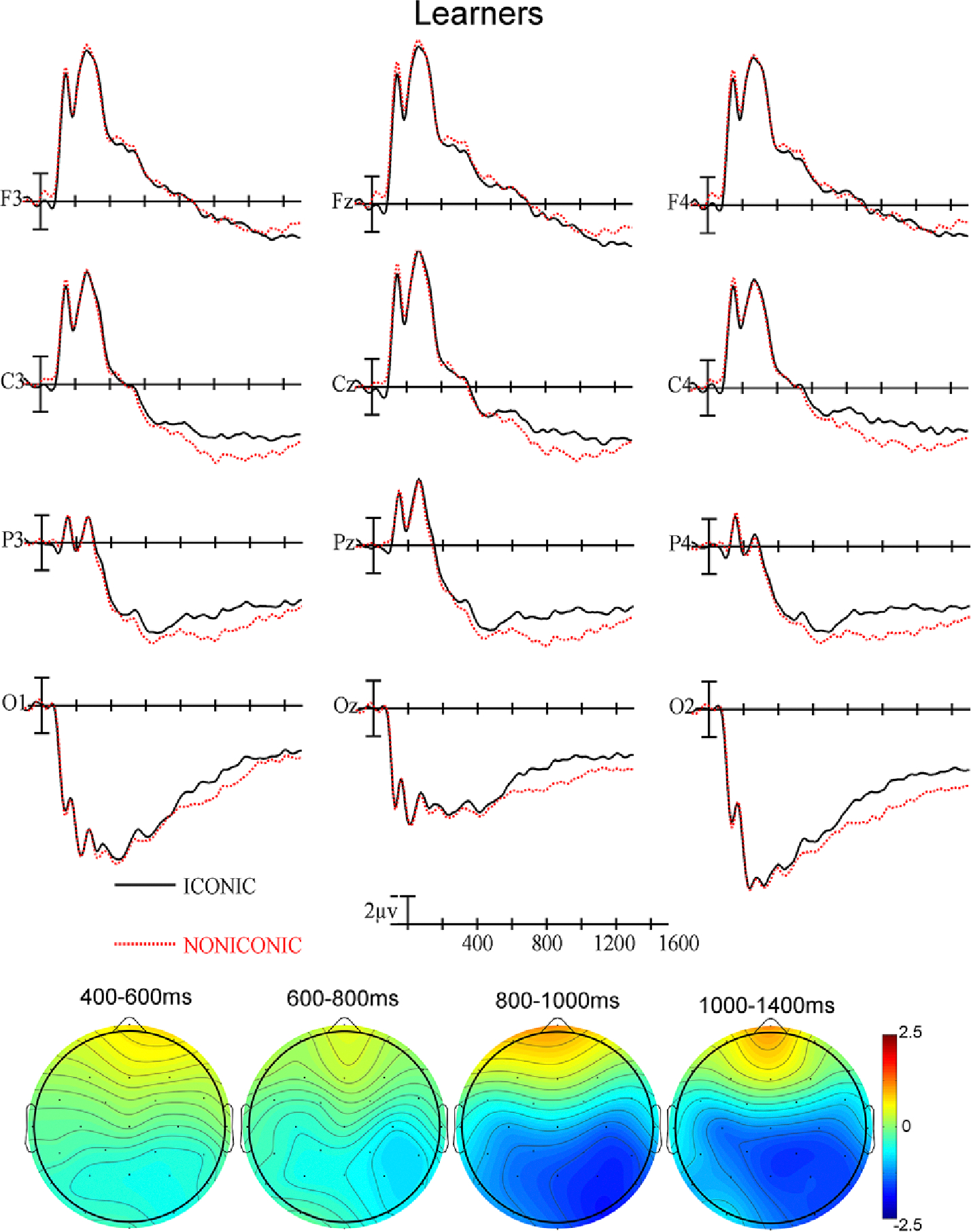
(Top) ERPs for learners at the 12 electrode sites used in the ANOVAs. (Bottom) Voltage maps were formed by subtracting iconic signs ERP trial data from non-iconic signs ERP trial data in the four latency ranges.

**Figure 4.**
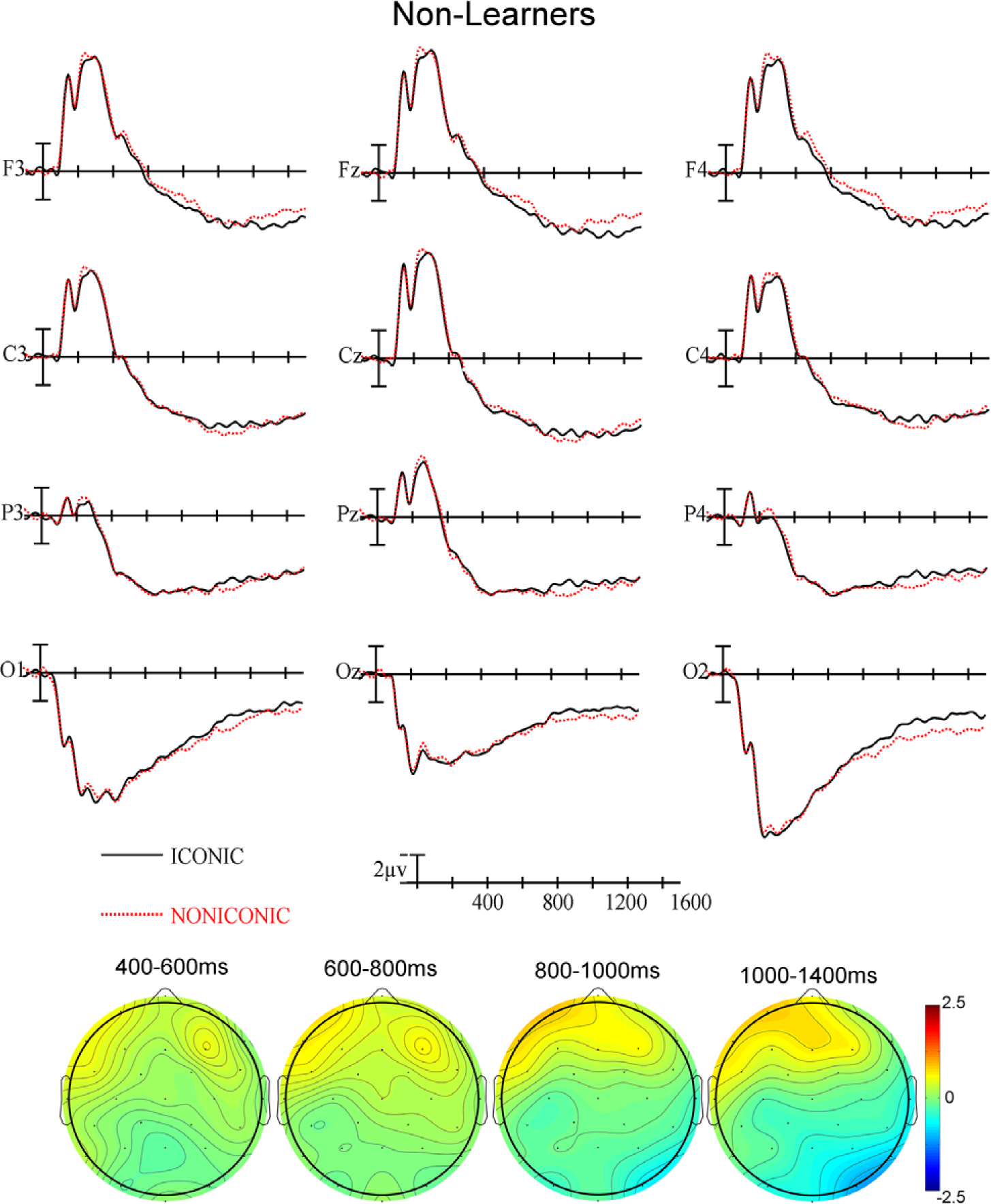
(Top) ERPs for non-learners at the 12 electrode sites used in the ANOVAs. (Bottom) Voltage maps were formed by subtracting iconic signs ERP trial data from non-iconic signs ERP trial data in the four latency ranges.

For the learners, there was a significant main effect of Iconicity (F(1,31) = 7.16, *p* = .0118, η_p_^2^ = .1876) as well as a significant interaction between Iconicity and Anteriority (F(3,93) = 9.07, *p* < .0001, η_p_^2^ = .2264) with iconic signs showing greater posterior negativity than non-iconic signs (see Figure 3). For the non-learners, there were no significant effects of iconicity (all *ps* > .05 – see Figure 4).

#### 1000–1400 ms time epoch:

In the last epoch, there was a significant main effect of Iconicity (F(1,62) = 8.75, *p* = .0044, η_p_^2^ = .1237) – iconic signs continue to show greater negativity than non-iconic signs, as well as a significant interaction between Group and Anteriority (F(3,186) = 4.89, *p* = .0234, η_p_^2^ = .0731) – the effect seen in the windows above continues in this epoch: learners exhibited greater negativity anteriorly and greater positivity posteriorly. In addition, there was a significant two-way interaction between Group and Iconicity (F(1,62) = 4.64, *p* = .0351, η_p_^2^ = .0697); therefore, we conducted ANOVAs for each group separately.

For the learners, there was a significant main effect of Iconicity (F(1,31) = 10.83, *p* = .0025, η_p_^2^ = .2589). There was a two-way interaction between Iconicity and Anteriority (F(3,92) = 7.85, *p* = .0001, η_p_^2^ = .202), with iconic signs showing greater posterior negativity than non-iconic signs. There was also a three-way interaction between Iconicity, Laterality and Anteriority (F(6,186) = 4.35, *p* = .0004, η_p_^2^ = .1231), indicating that iconicity effect was more lateralized to the right.

For the non-learners, there was no main effect of iconicity – in contrast to the learners, but the interactions between iconicity and scalp distribution patterned similarly to the learners. Specifically, there was a significant two-way interaction between Iconicity and Anteriority (F(3,93) = 6.03, *p* = .0109, η_p_^2^ = .1628) and a three-way interaction between Iconicity, Laterality and Anteriority (F(6,186) = 3.9, p = .0054, η_p_^2^ = .1119).

### 3.3. P3 component analysis

**Figure 5.**
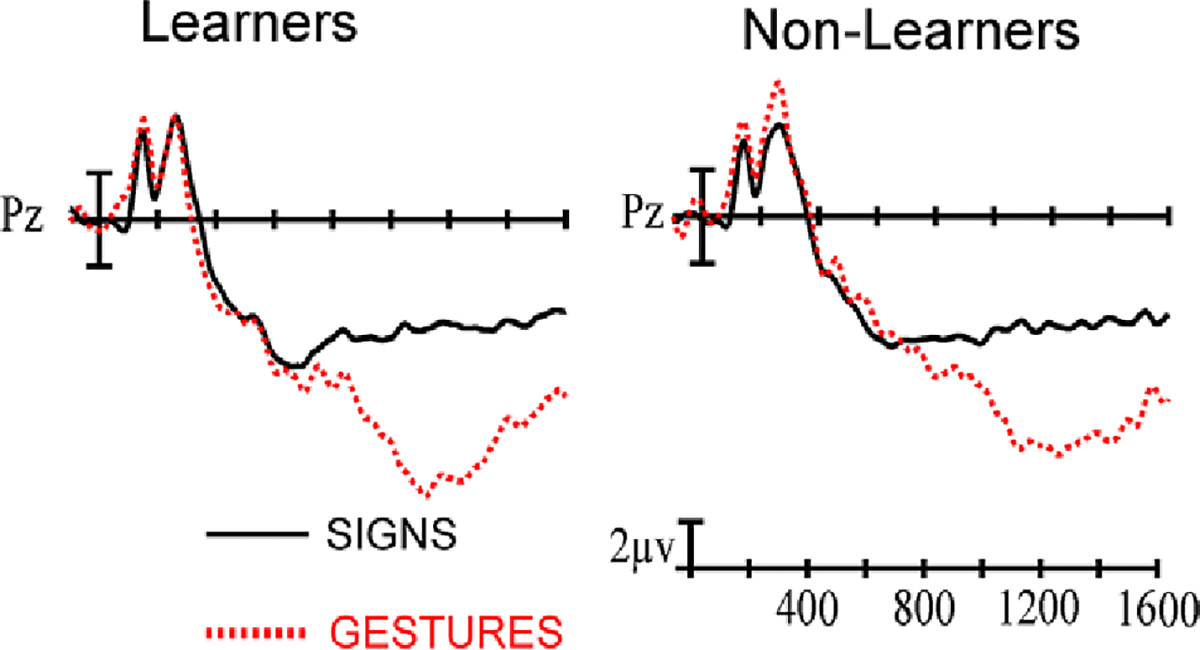
ERPs for learners and non-learners for the P3 component at the Pz electrode site, comparing responses to gestures (red) and iconic signs (black).

As anticipated, there was a main effect of Stimulus type (F(1,62) = 120.15, *p* < .001, η_p_^2^ = .6596), with the grooming gestures eliciting a larger posterior positivity (P3) than the signs. Importantly, there were no interactions between Group and Stimulus type (all *ps* > .36), indicating that the learners and non-learners were performing the gesture detection task similarly (See Figure 5).

## 4. Discussion

If sign-naïve people extract meaning from highly iconic (transparent) signs, then these signs should elicit greater negativity than non-iconic signs, particularly in the N400 time window. The N400 window for sign stimuli presented as full videos (i.e., the video starts with the signer’s hands in rest position) is defined as 800–1000 ms because the average sign onset within the video for this study was 578 ms – note that Emmorey et al. (2022) found that N400 priming effects were very similar when the ERPs were time-locked to video onset or to sign onset. We hypothesized that if meaning processing is automatic for transparent signs, then both learners and non-learners should show an iconicity effect (i.e., greater negativity for iconic than non-iconic signs). However, if only meaning-promoting tasks (e.g., word-sign matching) elicit an iconicity effect, then neither group should show a difference between sign types because our gesture-detection task did not require semantic processing. Finally, if an intent to learn signs is necessary to promote meaning processing, then only the learner group who were expecting to learn ASL signs should show an iconicity effect. The results support the last hypothesis.

The learners exhibited greater negativity for iconic than non-iconic signs in the N400 window (see Figure 3), but the non-learners did not (see Figure 4). The highly iconic and transparent signs presented in this study are likely to resemble the gestures that hearing people produce when pantomiming the concept conveyed by the sign, e.g., tracing a circle in the air for the concept ‘circle’ or miming drinking from a cup for the concept ‘drink.’ Our finding that non-learners did not exhibit an N400 effect for these gesture-similar signs indicates that form-meaning associations are not automatically extracted from gestures/signs when the task does not promote semantic processing. Thus, the meaning of even highly iconic signs is not processed automatically or unconsciously as has been found for iconic co-speech gestures (Gurney et al., 2013; Johnstone et al., 2023; Cassell et al., 1999; Kelly et al., 1999). However, co-speech gestures differ from isolated gestures/iconic signs because they are automatically integrated with the accompanying speech (Holle & Gunter, 2007: Özyürek et al., 2007). Co-speech gestures occur frequently and are argued to be processed as an integral part of language (e.g., McNeill, 1992). We suggest that gestures/iconic signs presented in isolation may be processed more like words in an unfamiliar language if there is no context to support semantic interpretation. In contrast to the non-learner group, the learner group was expecting to acquire the meanings of signs as part of a new lexicon, and they may have thus been sensitive to the “manual cognate” status of these signs with pantomimic gestures (Ortega et al., 2020).

The non-learners were only weakly sensitive to iconicity in the late time window (1000–1400 ms), which generally followed sign offset. We suggest that the non-learners may have recognized the meaning of at least some of the highly iconic signs, but they were much slower to do so than the learners. The learner group was motivated to identify ASL signs that they would be learning, while the non-learner group was primarily looking for target grooming gestures and may have been much less focused on the sign stimuli. We suggest that the late, weak effect of iconicity for the non-learners reflects less automatic, post-stimulus assessment of meaning.

Learners exhibited a large anterior negativity and posterior positivity throughout the recording compared to non-learners (see Figure 2). Even before sign onset, when we would not expect participants to be able to extract semantic information about the signs, learners were showing a strong neural difference compared to non-learners. Previous research has shown that when participants are exerting attention or using top-down processing, they demonstrate strong prefrontal cortex activation (Miller & Cohen, 2001). ERP studies in auditory language processing have found greater negativity when participants attended to a stimulus than when the stimuli were unattended (Hansen & Hillyard, 1980; Woldorff & Hillyard, 1991). We interpret the strong anterior negativity in the learners compared to the non-learners to be evidence that the learners were attending more to the stimuli than the non-learners. Greater negativity for learners was observed in the earliest time window (400–600 ms) before sign onset, indicating that this group difference was not due to variation in semantic processing.

The P3 component has been consistently shown to be affected by task (stronger for stimuli related to the task; Squires et al., 1975) and to exhibit greater amplitude for infrequent stimuli (Courchesne et al., 1975), particularly in paradigms where participants must make explicit decisions (e.g., Nieuwenhuis et al., 2005). Thus, we anticipated strong P3 effects for grooming gestures compared to the sign stimuli because gestures were presented on <15% of trials, and participants were specifically asked to detect them. The amplitude of the P3 component can be used as a measure of whether the learners and non-learners were performing the task in a similar manner, i.e., a group difference in the response to the task would be evident as larger or smaller P3 waves. However, we did not find any group differences in the P3 component or any interactions between Group and Stimulus Type, indicating both groups were performing the task similarly. We also found no differences between the learners and non-learners in task accuracy or number of false alarms. Both groups were equally able to discriminate between signs and grooming gestures.

Overall, the learners exhibited an iconicity effect in the N400 time window, whereas the non-learners did not. Thus, even before learning any signs and when performing a task that did not require semantic processing, participants in the learner group nonetheless attempted to extract meaning from the signs that were presented. In contrast, the participants in the non-learner group did not quickly or easily recognize the meaning encoded in the form of highly transparent ASL signs. The learners also showed greater frontal negativity for all signs throughout each epoch compared to the non-learners. This neural difference was observed even before sign onset, suggesting that the learners were attending more to the sign stimuli when performing the gesture-detection task. We conclude that comprehending the form-meaning mapping of highly iconic signs that resemble gestures does not occur automatically and requires attention and motivation.

## Supplementary Material

Supplementary Material

## Figures and Tables

**Table 1.** Means and standard deviation for false alarms and accuracy for the learner and non-learner groups in the gesture detection task

	Learners	Non-learners
Iconic false alarm	M = 18%, SD = 20%	M = 15%, SD = 19%
Non-iconic false alarm	M = 5%, SD = 5%	M = 5%, SD = 5%
Total false alarm	M = 12%, SD = 11%	M = 10%, SD = 11%
Accuracy	M = 92%, SD = 11%	M = 91%, SD = 11%

## Data Availability

The stimuli and data that support these findings are available at Open Science Framework at https://osf.io/7avju/

## Highlights

- Hearing non-signers viewed highly iconic and non-iconic ASL signs
- ERPs were collected during a grooming gesture detection task
- One group (learners) expected to learn ASL signs, while the other group did not
- Only learners showed a larger N400 to iconic than non-iconic signs
- Semantic processing of highly iconic signs requires attention

## Footnotes

1 By convention, ASL signs are glossed with the nearest English translation in upper case. Hyperlinks for the sign glosses link to videos of the signs from the ASL-LEX database (Caselli et al., 2017; Sehyr et al., 2021).
