# Supplementary material for: Semantic processing of iconic signs is not automatic: Neural evidence from hearing non-signers
Source: Biling (Camb Engl). Author manuscript; Available in PMC 2025 Oct 17. (PMC12530573; doi:10.1017/s1366728924001093)
Supplement: Supplementary Material [file NIHMS2056463-supplement-Supplementary_Material.docx]

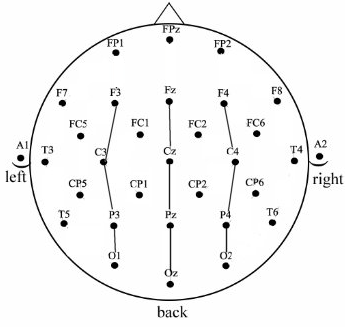


**Supplementary Materials Figure 1**. Electrode montage - sites used in analyses are connected by lines.
